# The Effect of Home and In-Office Bleaching on Microhardness and Color of Different CAD/CAM Ceramic Materials

**DOI:** 10.3390/ma15175948

**Published:** 2022-08-28

**Authors:** Ruwaida Z. Alshali, Mohammed A. Alqahtani

**Affiliations:** 1 Oral and Maxillofacial Prosthodontics Department, Faculty of Dentistry, King Abdulaziz University, Jeddah 21589, Saudi Arabia; 2 Prosthetic Dental Science, College of Dentistry, King Saud University, Riyadh 11545, Saudi Arabia

**Keywords:** bleaching, microhardness, color, ceramics, CAD/CAM

## Abstract

The aim of this study is to assess the effect of different bleaching agents on microhardness and color of CAD/CAM ceramics including IPS e.max CAD (lithium disilicate), VITA ENAMIC (polymer-infiltrated ceramic), and Celtra Duo CAD (zirconia-reinforced lithium silicate). Materials’ samples were divided into three groups (n = 10) and each received a different bleaching treatment; 20% carbamide peroxide, 35% carbamide peroxide, and 40% hydrogen peroxide. A fourth group was stored in water acting as a control. Vickers microhardness and spectrophotometric color measurements were taken at baseline and after bleaching. IPS e.max CAD showed a significant reduction (about 14%), while VITA ENAMIC showed a significant increase (about 78%) in microhardness after bleaching (*p* ˂ 0.001). Celtra Duo CAD did not demonstrate a significant change in microhardness (*p* ≥ 0.609). The color difference (ΔE_ab_) after bleaching was 0.29 (±0.08), 2.84 (±0.64), and 1.99 (±0.37) for IPS e.max CAD, VITA ENAMIC, and Celtra Duo CAD, respectively. It could be concluded that the effect of bleaching on color and microhardness was mainly material-dependent. Bleaching significantly affected the microhardness of IPS e.max CAD and VITA ENAMIC. The color difference was within the clinically imperceptible range for IPS e.max CAD, while VITA ENAMIC and Celtra Duo CAD demonstrated perceptible color change.

## 1. Introduction

The use of dental bleaching agents to whiten discolored teeth is gaining high popularity because of their effectiveness and conservation of tooth structure compared to restorative treatment [1]. Dental bleaching agents are primarily based on hydrogen peroxide (HP), which is applied directly during in-office bleaching, or in the form of carbamide peroxide (CP), which is used in home-bleaching products. Hydrogen peroxide results in the bleaching of the dental hard tissues through the release of free radicals that diffuse into the dental enamel and dentin tissues degrading the large organic stain molecules (chromophores) into smaller, white, colorless molecules through the conjugation of double bonds [2]. Although bleaching is considered a safe procedure, it could induce changes in enamel and dentinal tissues at the microstructural level resulting in transient tooth sensitivity. Bleaching with 10% CP was shown to result in a decrease in enamel microhardness and an increase in its surface roughness [3]. The addition of remineralizing nanoparticles in the bleaching products was shown to improve the resistance of enamel to surface changes and was suggested as a possible approach for decreasing transient sensitivity following bleaching [4].

Although bleaching agents are intended for treating only the natural dental hard tissues, they can inadvertently come into contact with dental materials restoring the teeth in the esthetic zone. The effect of HP-based bleaching agents on the physical properties of various dental restorative materials has been widely assessed, including their effect on surface roughness, gloss, color, microhardness, and flexural strength. Tooth-colored restorative materials such as glass ionomers, resin-modified glass ionomers [5], and polyacid-modified composites [6] were shown to be negatively affected by bleaching procedures, while resin-composites showed variable results which were mainly material- and time-dependent [7]. Concerning ceramic materials, the effects of bleaching on surface roughness, color, and microhardness are not consistent among studies and appear to be dependent on the type of ceramic material, the method of surface treatment (whether autoglazed, overglazed, or polished), and the concentration and application time of the bleaching agent [7]. Monolithic glazed zirconia was recently investigated and showed no significant changes in surface roughness and color, but demonstrated a significant reduction in microhardness after HP in-office bleaching [8].

Dental ceramics are currently fabricated using CAD/CAM technology which facilitated the chair-side production of high-standard all-ceramic restorations (crowns, onlays, and veneers) with reduced porosity and greater strength compared to conventional fabrication techniques [9]. Chair-side CAD/CAM ceramic materials include leucite-reinforced ceramics, lithium disilicate, and hybrid ceramic materials containing cross-linked polymeric matrices such as resin nano-ceramics and polymer-infiltrated ceramics [10]. More recently, zirconia-reinforced lithium silicate (lithium silicate/phosphate) has been introduced which requires no sintering, in addition to the high-speed sintering of translucent zirconia [11]. A few studies have assessed the effect of bleaching on the surface properties of some of these recently introduced CAD/CAM materials [12,13,14,15]; however, more data are required regarding the effect of different concentrations of bleaching agents applied on a wider range of CAD/CAM ceramic materials.

Surface microhardness is defined as the material’s resistance to permanent indentation. It is a critical property for a restorative material that reflects its mechanical strength [16], its resistance to wear, and its abrasiveness to opposing dental tissues and restorative materials [17]. A change in surface microhardness of a material indicates structural degradation or solubility that would be associated with a reduction in the material’s strength and mechanical performance [18]. Color stability is another property that is required during a restoration life-time to resist staining and maintain aesthetics. Tooth-colored materials should demonstrate high color stability against food and beverages and dental hygiene and bleaching agents that come into direct contact with teeth and restorations. These substances and agents may either directly stain a restoration or affect its structure. Color stability depends on surface smoothness, chemical stability, and the resistance of the dental material to chemical softening and diffusion [19]. Color assessment can be performed more reliably by instrumental methods compared to visual assessment. Instrumental techniques for color measurement include the use of colorimeters, spectrophotometers, and digital imaging techniques, with spectrophotometers showing the most accurate results [20].

The aim of this study is to assess the effect of different bleaching agents, including 40% hydrogen peroxide (40% HP), 35% carbamide peroxide (35% CP), and 20% carbamide peroxide (20% CP) on microhardness and color of different CAD/CAM ceramics. The ceramic materials included lithium disilicate, polymer-infiltrated ceramic, and zirconia-reinforced lithium silicate. The null hypotheses to be tested were:

1. There is no significant change in microhardness after bleaching the different ceramic materials using the different bleaching agents;
2. The extent of change of microhardness and color after bleaching is not significantly influenced by the type of ceramic material;
3. The extent of change of microhardness and color after bleaching is not significantly influenced by the type of bleaching agent.

## 2. Materials and Methods

### 2.1. Test Materials

Materials included three CAD/CAM ceramics: IPS e.max CAD, VITA ENAMIC, and Celtra Duo CAD, which represent lithium disilicate, polymer-infiltrated ceramic, and zirconia-reinforced lithium silicate, respectively. Bleaching materials included 40% hydrogen peroxide in-office bleaching and two carbamide peroxide home-bleaching agents with 20% and 35% concentration. Table 1 summarizes information regarding the ceramic and bleaching materials’ composition and manufacturers’ details.

### 2.2. Sample Preparation and Experimental Design

**Figure 1:**
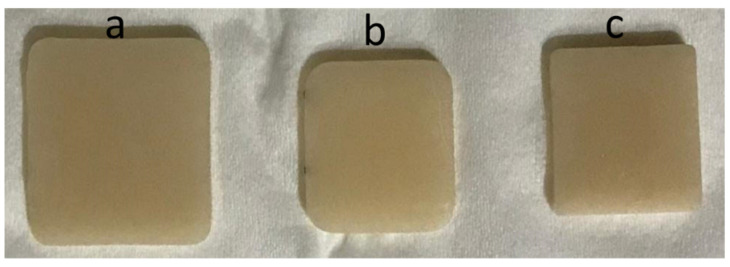
The finished and polished ceramic samples; (**a**) IPS e.max CAD, (**b**) Celtra Duo CAD, and (**c**) VITA ENAMIC.

Specimens of the three ceramic materials were prepared (40 specimens for each material) by sectioning the ceramic CAD/CAM blocks using a precision low-speed saw with water coolant (TECHCUT 4, Allied High Tech Product, Pacifica Place, Rancho Dominguez, CA, USA). The ceramic blocks were sectioned into rectangular slices with an approximately 2-mm thickness (Figure 1) giving a total of 120 samples (40 samples of each material). A thickness of 2 mm was determined to minimize the effect of background color and translucency on the computed color. A recent study showed that ceramic thickness not less than 2 mm is required to mask the color of most backgrounds [21].

The edges of the ceramic samples were refined using high-speed yellow and red wheel diamond burs with water (Meisinger, Neuss, Germany). One surface of each specimen was polished using a grinder-polisher appliance (MetaServ 250, BUEHLER, Waukegan Road, Lake Bluff, IL, USA) with water and a speed of 250 rpm. During the polishing procedure, each sample was manually held for 1 min against a fitted silicon carbide grinding paper (MicroCut Discs, PSA Backed, Buehler, USA) with three different grit sizes; P60 (190 µm), P400 (35 µm), and P2500 (8 µm). The grinding papers were used in sequence until the surface of a sample was visibly glossy. The chosen grit sizes allowed simulation of rubber polishing systems (medium, extra fine, and super fine) that are used clinically to produce acceptable surface roughness of less than 0.2 Ra value [22]. All finishing and polishing procedures were conducted by a single operator who was trained to apply a constant force using an electronic digital scale before the actual sample preparation was performed.

Samples of IPS e.max CAD were then crystallized in a furnace at a temperature of 850 °C for about 20 min according to the manufacturer’s recommended crystallization programme (Programat P310, Ivoclar Vivadent, Schaan AG, Liechtenstein). All materials’ samples were then stored in distilled water for 24 h in an incubator at 37 °C. Baseline measurements of microhardness and color for the polished surfaces of all specimens were taken. 

Samples of each ceramic material were randomly divided into four groups (n = 10). Three groups received three different bleaching treatments, and the fourth group was not bleached to act as a control. The control group of each material was kept stored in distilled water at 37 °C, while the other three groups received treatment for the polished surfaces using the specified bleaching agents; 20% CP, 35% CP, and 40% HP. 

Each bleaching agent was applied according to the manufacturer’s instructions. For the 20% CP and 35% CP, the bleaching gel was applied for 4 h per day and 60 min per day, respectively, for 7 days. After each application, the specimens were rinsed with water for 10 s and stored in distilled water at 37 °C until the next application, simulating the intra-oral condition. The 40% HP bleaching gel was applied twice, each turn for 20 min. After the first application, the specimens were rinsed with distilled water for 10 s and dried, followed by the second application. All bleaching agents were spread on the polished sample surfaces with a brush, completely covering the surface with a uniform thickness of material (0.5–1.0 mm). The bleaching treatment was performed by a single operator at room temperature (25 °C) and the samples were then immediately stored at 37 °C for the specified bleaching periods. 

A second measurement of microhardness and color was taken for the control and experimental groups. Samples were cleaned with a compressed water spray for 10 s and dried with compressed air before analyses.

### 2.3. Microhardness and Color Measurements

**Figure 2:**
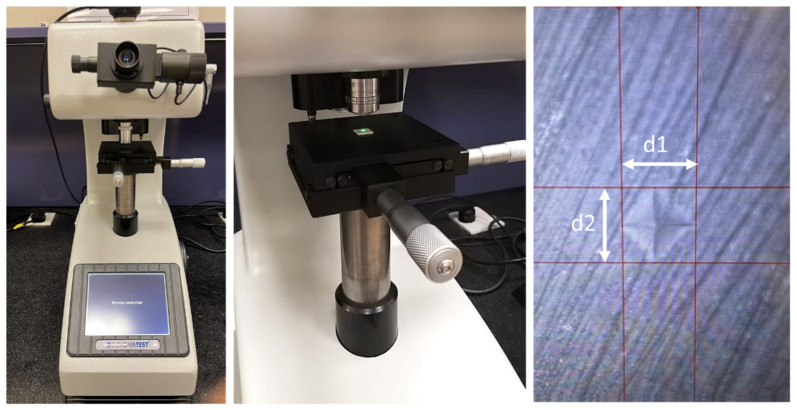
The microhardness testing device used in the study (on the left). The picture in the middle shows more closely the sample positioning table with a ceramic sample in place. The picture on the right side shows Vickers indentation of IPS e.max CAD before bleaching at 20× magnification. The edges of both diagonals are marked to determine their length followed by automatic calculation of the Vickers microhardness number according to the formula: Vickers microhardness number = 1.854 F/d^2^, where F is the applied load in kgf and d is the average diagonal length of the indentation measured in mm [24].

Vickers microhardness was assessed at the center of each sample using a microhardness testing device (INNOVA*TEST* Europe BV, Borgharenweg 140, 6222 AA Maastricht, The Netherlands). The testing was performed at 200 gf load and 10 s dwell time as recommended by the American Society for Testing and Materials (ASTM) [23]. The applied load produced Vickers indentations with diagonals not less than 20 μm in length so that the edges of the indents were clearly visible under magnification and could be precisely measured (Figure 2). 

To improve accuracy, three readings for each sample were taken. To avoid measuring areas distorted by previous indentations, the distance between the centers of two Vickers indentations was at least two and a half times the diagonal length of the indentations, according to the specification of the ASTM. 

Color measurements were performed according to ISO/TR 28642 [25] using a LabScan XE dual-beam spectrophotometer (Hunter Associates Laboratory Inc., 11491 Sunset Hills Road, Reston, VA 20190, United States). The device had a 5-mm diameter measuring area. The device operated with a pulsed Xenon lamp light source that was filtered to approximate D65 daylight (in the 400–700 nm spectral range). Samples were illuminated at 0° and viewed circumferentially at 45° simulating the viewing angle of the human eye. The reflected light was collected using a 15-station fiberoptic ring. The measurement time was ˂3 s with a 10 nm optical resolution. The instrument was calibrated using black glass and white tile standards every 4–8 min of use. The color readings were obtained as the L*a*b* coordinates of color; L* represented the color in the black-white axis, a* represented the red-green axis, and b* represented the yellow-blue axis. 

**Figure 3:**
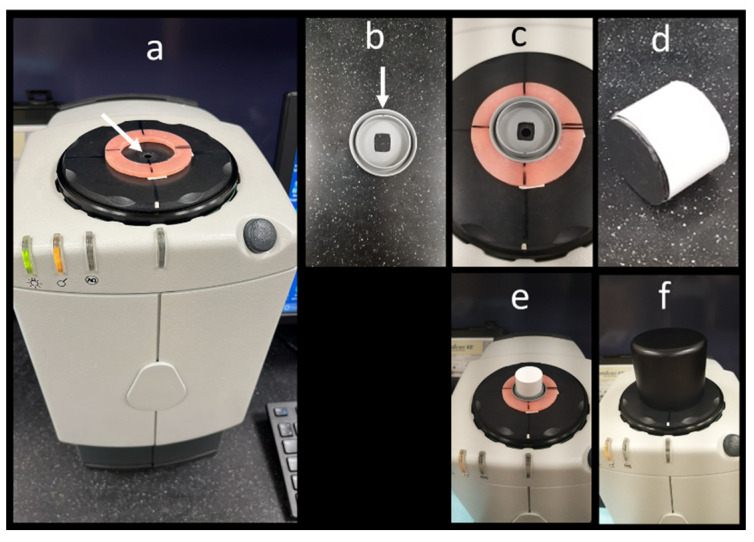
The spectrophotometer device used for color assessment: The white arrow points to the 5-mm diameter measuring window (**a**); A customized jig is fabricated for each material with a rectangular opening with similar dimensions to that of the specimen. A notch at the periphery (arrow) was used to standardize positioning the sample during measurement (**b**); The jig is placed within an acrylic ring fixed around the measuring window. The acrylic ring has four black line markings at 12-, 3-, 6-, and 9 o’clock positions. Three readings for each sample were taken by aligning the notch with the 12-, 3-, and 6 o’clock positions (**c**); A cylinder with a black surface is used as a customized black background (**d**); The cylinder is fitted inside the jig with the black end placed against the sample (**e**); A black cover is placed over the customized background and jig to block ambient light from reaching the measuring window when readings are taken (**f**).

Three measurements were taken for each sample and the average value was calculated. A customized jig was used to centralize each sample in a repeatable position against the spectrophotometer measuring area (Figure 3). Measurements were performed against a black background simulating the darkness of the oral cavity. The L*, a*, and b* values of the black background were 19.43 (±0.50), 0.10 (±0.07), and −0.74 (±0.14), respectively. 

The color difference (ΔE_ab_) was calculated using the following CIE Lab color difference formula:

ΔE_ab_ = [(ΔL*)^2^ + (Δa*)^2^ + (Δb*)^2^]^1/2^ (1)

where:

ΔL* = L* (after bleaching) − L* (before bleaching)

Δa* = a* (after bleaching) − a* (before bleaching)

Δb* = b* (after bleaching) − b* (before bleaching)

### 2.4. Statistical Analysis

The sample size calculation for ΔE_ab_ was based on a standard deviation value of 0.8, a minimum expected difference between comparison groups of 1.22 [26], and 95% study power. The sample size calculation for microhardness was based on a standard deviation value of 5, a minimum expected difference between comparison groups of 10, and 95% study power. A sample size of 10 was determined to be adequate for assessing differences in both microhardness and ΔE_ab_. The data were analyzed using IBM SPSS Statistics software version 20 for Windows (IBM Corporation, Armonk, NY, USA). The normality of the data was assessed using the Shapiro-Wilk test. Data for microhardness before and after bleaching and the percentage hardness change data in most of the groups were normally distributed; thus, parametric tests were used. These included paired sample t-tests to compare microhardness before and after bleaching, and one-way ANOVA with Tukey post hoc testing to compare the percentage change in microhardness between the different bleaching groups and different ceramic materials. Data for ΔE_ab_ were not normally distributed in most of the groups; thus, an independent-samples Kruskal-Wallis test was performed to compare ΔE_ab_ between the different bleaching treatments and ceramic materials. Pairwise comparisons were performed using a Mann-Whitney U test followed by Bonferroni correction for multiple tests. All tests were conducted at a significance level of 5%.

## 3. Results

**Figure 4:**
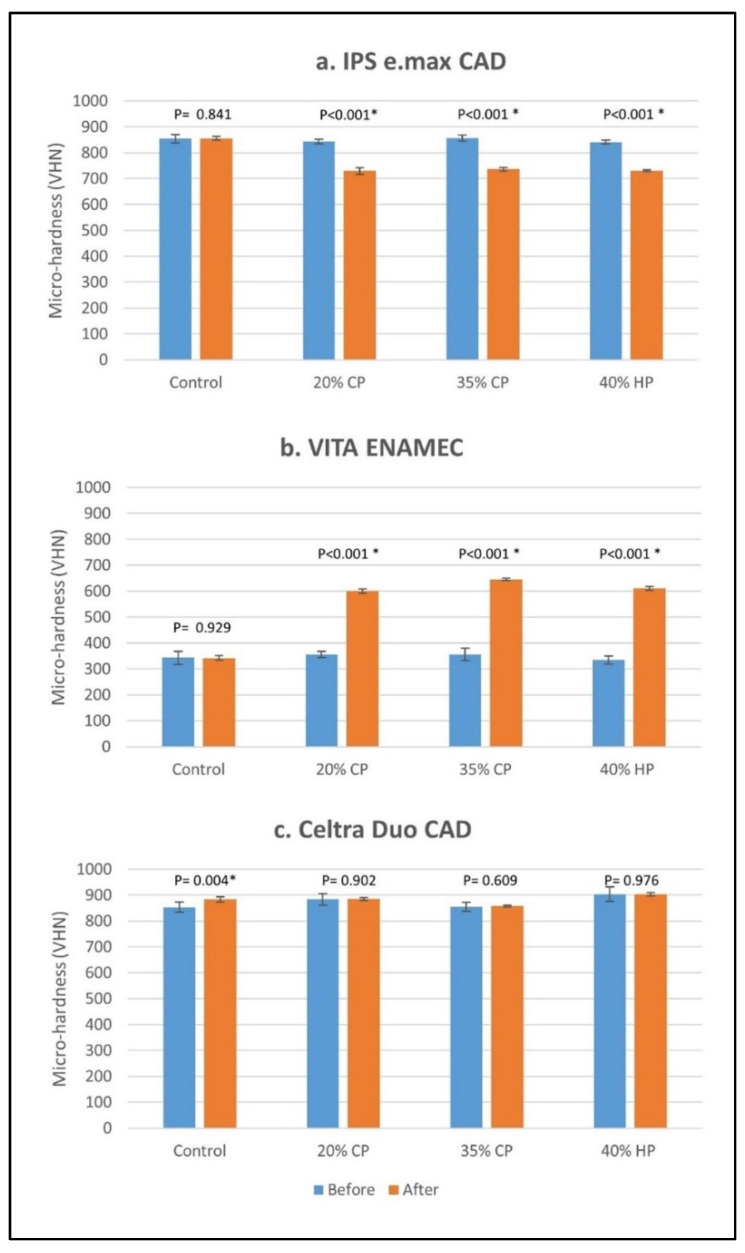
Vickers microhardness of the different ceramic materials before and after bleaching. Significant differences between values before and after bleaching are denoted with asterisk sign (α = 0.05). CP and HP stand for carbamide peroxide and hydrogen peroxide, respectively.

The mean microhardness values for the control groups before bleaching were 853.82 (±16.89) for IPS e.max CAD, 342.79 (±25.69) for VITA ENAMIC, and 853.68 (±19.46) for Celtra Duo CAD. For IPS e.max CAD, microhardness significantly decreased after bleaching in all bleaching groups (*p* ˂ 0.001) while the control group was not affected (*p* = 0.841). For VITA ENAMIC, microhardness significantly increased after bleaching in all bleaching groups (*p* ˂ 0.001) but no significant change was shown in the control group (*p* = 0.929). For Celtra Duo CAD, there was no significant change in microhardness in all bleaching groups (*p* ≥ 0.609), however, the control group showed a significant increase in microhardness (*p* = 0.004) (Figure 4). 

The different ceramic materials showed significantly different percentage changes in microhardness and ΔE_ab_ after bleaching (*p* ˂ 0.001). The percentage change in microhardness after bleaching ranged from −13.96% to −13.06% for IPS e.max CAD, from 68.74% to 82.74% for VITA ENAMIC, and from 0.13% to 0.41% for Celtra Duo CAD. The mean ΔE_ab_ in the bleaching groups ranged from 0.21 to 0.37 for IPS e.max CAD, from 2.38 to 3.58 for VITA ENAMIC, and from 1.77 to 2.41 for Celtra Duo CAD. 

For IPS e.max CAD, there were no significant differences in the percentage change of microhardness between the different bleaching groups (*p* = 0.539), but they were all significantly different than the control group (*p* ˂ 0.001). In VITA ENAMIC, 20% CP showed a significantly lower percentage change in microhardness compared to 35% CP and 40% CP bleaching groups (*p* ≤ 0.011), but all bleaching groups showed a significantly higher percentage change in microhardness compared to the control (*p* ˂ 0.001). Concerning Celtra Duo CAD, there were no significant differences between different bleaching groups (*p* = 0.996). The percentage change in microhardness of the control group was 3.53%, which was significantly higher than the bleaching groups (*p* ≤ 0.046) except for 35% CP (*p* = 0.074). 

There was no significant difference in ΔE_ab_ between the different bleaching groups, including the control group for IPS e.max CAD (*p* = 0.206), and Celtra Duo CAD (*p* = 0.050). For VITA ENAMIC, all bleaching groups showed a significantly higher ΔE_ab_ compared to the control group (*p* ≤ 0.031) but there were no significant differences between the different bleaching groups (*p* ≥ 0.279). Table 2 summarizes the data and statistics regarding the percentage change in microhardness (%) and ΔE_ab_ for the different ceramic materials and bleaching groups.

## 4. Discussion

IPS e.max CAD (lithium disilicate) showed a significant reduction in microhardness (about 14%) while VITA ENAMIC (polymer-infiltrated ceramic) showed a significant increase in microhardness (about 78%) after bleaching with all different agents. Celtra Duo CAD (zirconia-reinforced lithium silicate) did not demonstrate a significant change in microhardness after bleaching. Thus, the first null hypothesis was partially rejected. The percentage change in microhardness and ΔE_ab_ were significantly influenced by the type of ceramic material; thus, the second null hypothesis was rejected. The percentage changes in microhardness and ΔE_ab_ were not significantly influenced by the type of the bleaching agent (except 20% CP which showed a significantly lower percentage change in microhardness for VITA ENAMIC); thus, the third null hypothesis was partially rejected. 

The bleaching protocol used in the current study was performed following the manufacturer’s recommendations. For the CP home-bleaching products, 7-day bleaching was considered adequate to achieve noticeable results, while longer bleaching periods were used in other studies. Although increasing the concentration of the bleaching agent was shown to be associated with an increased surface roughness in feldspathic ceramics in a previous study [27], different concentrations of bleaching agents did not show significant differences in terms of microhardness and color change in any of the restorative materials in the current study. This finding is consistent with a previous study that showed no differences in microhardness change associated with different CP concentrations (10% and 16% CP) for different materials, including feldspathic porcelain, microfilled resin composite, and a resin-modified glass ionomer [28]. Only in the case of VITA ENAMIC, 35% CP and 40% HP showed a significantly greater change in microhardness compared to 20% CP. 

The current study showed a significant reduction in microhardness of IPS e.max CAD after bleaching (about 14% reduction in microhardness), a finding that has not been shown previously except when this material was immersed in acidic solutions [29]. A previous study showed a reduction in microhardness of lithium disilicate after bleaching with 35% HP for three sessions (each session consisted of three 15-min applications) but the reduction was statistically insignificant [13]. An insignificant reduction in microhardness of polished and glazed lithium disilicate after bleaching was shown in another study, in which 16% CP was applied for 6 h per day for 7 days [12]. Self-glazed feldspathic porcelain in a previous study showed a significant reduction in microhardness (15% reduction) when bleached with 10% and 16% CP for 8 h per day for 30 days [28]. This decrease in microhardness could be related to the reduction in surface SiO_2_ content which forms the glassy matrix of all glassy ceramic materials, as was shown by the energy dispersive X-ray microanalysis following the bleaching of feldspathic porcelain [30]. Reduction in silicon, potassium, and aluminum was also shown by elemental analysis of different types of ceramics immersed in acidic drinks [31], suggesting that ceramics are susceptible to low pH and oxidizing solutions, resulting in their dissolution and chemical degradation. Surface roughness analysis of glazed lithium disilicate has shown a significant increase in surface roughness after bleaching with 16% CP, which was confirmed with SEM imaging revealing erosion and increased surface porosity; a finding that corroborates with the reduction in surface hardness [12]. 

Regarding Celtra Duo CAD, microhardness was not affected by any of the applied bleaching agents. This finding suggests greater chemical stability of this material which could be attributed to an increase in the tetragonal zirconia crystalline content compared to IPS e.max CAD [32]. In the current study, the control group of Celtra Duo CAD exhibited a significant increase in microhardness (3.5%); however, this increase is considered insignificant from the clinical point of view and should have no implications. 

The current study showed that the microhardness in VITA ENAMIC significantly increased after bleaching (about a 78% increase in microhardness). This finding is consistent with the results of a recent study that showed an 11% increase in microhardness of VITA ENAMIC after bleaching with 35% HP [13]. This increase in microhardness of VITA ENAMIC after bleaching could be the result of degradation of the polymeric network in the material by the action of HP free radicals leaving the harder ceramic component exposed on the surface. Although this may appear as a positive change, it could have a negative effect on other surface properties such as an increase in surface roughness and changes in the material’s optical properties. A previous study assessing the effect of home and in-office bleaching on the surface roughness of two hybrid ceramic materials, including VITA ENAMIC, showed a significant increase in roughness [14]. Another study showed surface pitting of VITA ENAMIC on SEM images after bleaching with 6% HP and 15% CP [15]. These findings would explain the increase in surface microhardness after bleaching that was shown in the current study. 

Concerning color change, the results of the current study showed that IPS e.max CAD demonstrated a significantly lower ΔE_ab_ value compared to VITA ENAMIC and Celtra Due CAD. There is controversy in the literature regarding ΔE_ab_ values associated with clinically perceptible and acceptable color changes for restorative materials. A robustly designed multicenter study with different observer groups has shown that ΔE_ab_ 50:50% perceptibility and acceptability thresholds were 1.2 and 2.7, respectively [26]. Accordingly, the color change of IPS e.max CAD in the current study was below the perceptibility threshold, while the change of Celtra Duo CAD and VITA ENAMIC was perceptible but within the acceptable range. The only group that showed ΔE_ab_ beyond the acceptability threshold was the VITA ENAMIC treated with 40% HP. The result of the current study concerning the color change of IPS e.max CAD after bleaching is in agreement with a previous study [15]; however, ΔE_ab_ values for VITA ENAMIC were reported to be below the perceptibility level [14,15], which is contradictory to the findings of the current study. This could be attributed to differences in concentration, application time, and pH of the bleaching agents. Moreover, the discrepancy between study findings could be attributed to differences in the color measurement protocols. 

The perceptible color change of VITA ENAMIC and Celtra Duo CAD compared to the imperceptible change of IPS e.max CAD cannot be directly related to changes in microhardness, because microhardness and color describe independent surface properties (mechanical versus optical) that are influenced by different factors. However, this finding could more likely be attributed to changes in surface roughness after bleaching, which may affect the color through changing surface reflectivity [33]. A previous study confirmed that bleaching resulted in an increase in surface roughness, which was significantly greater in VITA ENAMIC compared to IPS e.max CAD [15]. Unfortunately, no data are available in the literature regarding the effect of bleaching on the surface roughness of Celtra Duo CAD. 

According to the findings of the current study, it is advisable to protect restorations made out of CAD/CAM lithium disilicate, polymer-infiltrated ceramic, and zirconia-reinforced lithium silicate during in-office and home bleaching procedures. The significant reduction in microhardness of IPS e.max CAD could negatively affect the mechanical performance and longevity of the restorations. The perceptible color change of VITA ENAMIC and Celtra Duo and the significant increase in microhardness of the former could be associated with an unfavorable increase in surface roughness, which increases biofilm adhesion and staining of these materials. Thus, polishing of VITA ENAMIC and Celtra Duo is advisable when color changes are observed after their exposure to bleaching agents. 

The current study has a number of limitations that should be addressed and considered in future research. One of the limitations is that the CIE Lab color-difference formula (ΔE_ab_) was used instead of the latest international standard CIEDE 2000 formula (ΔE_00_) recommended by the Commission International de l′Éclairage (CIE) [34]. Although the two formulas have been shown to give highly correlated color differences, the CIEDE 2000 formula is recommended for color assessment since it involves correction for the difference in computed and perceived color. The CIEDE 2000 formula links the computed color values to the Munsell color system that determines color according to the three parameters of value, hue, and chroma, which are based on the responses of the human eye, thus giving a more reliable assessment of color perceptibility and acceptability thresholds [35]. However, the CIE Lab color-difference formula is the most commonly used even in today’s research because of its simplicity compared to the CIEDE 2000 formula. This makes it possible to compare the findings of different studies. Another limitation of this study is that the effect of bleaching on stain susceptibility was not assessed, which is of high clinical relevance. This could be achieved by immersing the samples in different beverages after bleaching (such as coffee and cola) followed by spectrophotometric color measurements. Finally, it would be interesting to apply the bleaching agents over a longer time period and repeat the measurements of microhardness and color over multiple time points to assess whether the changes would be affected by time.

## 5. Conclusions

Within the limitations of the current study, the following can be concluded:

1. Bleaching treatments significantly affected the microhardness of IPS e.max CAD and VITA ENAMIC resulting in reduced microhardness in the former (−13.96% to −13.06%) and increased microhardness in the latter (68.74% to 82.74%.). The microhardness of Celtra Duo CAD was not affected by bleaching.
2. The 35% CP and 40% CP resulted in a greater percentage increase in microhardness compared to the 20% CP for VITA ENAMIC.
3. The color change was within the imperceptible range for IPS e.max CAD, while VITA ENAMIC and Celtra Duo CAD demonstrated perceptible color changes within the clinically acceptable range.

## Figures and Tables

**Table 1:** The composition and manufacturer details of the tested ceramic materials.

Trade Name	Type	Composition	Manufacturer	Batch Number/Shade
VITA ENAMIC	Hybrid ceramic	Fine structure feldspar ceramic (86% wt.): SiO_2_ (58–63%), Al_2_O_3_ (20–23%), Na_2_O (6–11%), K_2_O (4–6%), B_2_O_3_ (0.5–2%), CaO (˂1%), TiO_2_ (˂1%). Polymers (14% wt.): urethane dimethacrylate (UDMA) and triethylene glycol dimethacrylate (TEGDMA).	VITA Zahnfabrik, Bad Sackingen, Germany	78240/LT-A2 C14
IPS e.max CAD	lithium disilicate glass-ceramic	SiO_2_ (57.0–80.0%), Li_2_O (11.0–19.0%), K_2_O (0.0–13.0%), P_2_O_5_ (0.0–11.0%), ZrO_2_ (0.0–8.0%), ZnO (0.0–8.0%), Al_2_O_3_ (0.0–5.0%), MgO (0.0–5.0%), Coloring oxides: 0.0–8.0%.	Ivoclar Vivadent, Schaan AG, Liechtenstein	W06535/LT-A2 C14
Celtra Duo CAD	Zirconia-Reinforced Lithium Silicate	Lithium silicate, 10% zirconium dioxide (ZrO_2_).	Dentsply Sirona, West Clarke Avenue, Germany	16005398/LT-A2 C14
Opalescence PF 20% (20% CP)	Home bleaching	20% Carbamide peroxide, Potassium nitrate, 0.11% fluoride (1100 ppm), 20% water. (pH = 6.5)	Ultradent Product, West Ultradent Drive, South Jordan, USA	BH5W2
Opalescence PF 35% (35% CP)	Home bleaching	35% Carbamide peroxide, Potassium nitrate, 0.11% fluoride (1100 ppm), 20% water. (pH = 6.5)	Ultradent Product, West Ultradent Drive, South Jordan, USA	BHCF1
Opalescence Boost 40% (40% HP)	In-office bleaching	40% Hydrogen peroxide, 1.1% fluoride, 3% Potassium nitrate (supplied in two syringes) (Neutral pH)	Ultradent Product, West Ultradent Drive, South Jordan, USA	BHFRH

**Table 2:** Percentage change in microhardness (%) and ΔE_ab_ of different ceramic materials and bleaching groups. Values represent the mean and standard deviation (in parentheses). Similar lower case superscript letters represent non-statistically different values per column for each property. Similar superscript upper case letters represent non-statistically different values per row (α = 0.05). CP and HP stand for carbamide peroxide and hydrogen peroxide, respectively.

	IPS e.Max CAD	VITA ENAMIC	Celtra Duo CAD	*p*-Value
**Percentage change in microhardness (%)**				
Control	0.15 (1.85) ^a,A^	0.28 (8.05) ^a,A^	3.54 (2.99) ^a,A^	0.257
20% CP	−13.49 (1.60) ^b,A^	68.74 (6.57) ^b,B^	0.14 (2.31) ^b,C^	˂0.001
35% CP	−13.96 (1.45) ^b,A^	82.14 (12.68) ^c,B^	0.41 (2.22) ^a,b,C^	˂0.001
40% HP	−13.06 (0.91) ^b,A^	82.74 (7.77) ^c,B^	0.13 (3.41) ^b,C^	˂0.001
*p*-value	˂0.001	˂0.001	0.023	
**ΔE** **_ab_**				
Control	0.34 (0.13) ^a,A^	1.03 (0.37) ^a,B^	2.70 (0.67) ^a,C^	˂0.001
20% CP	0.37 (0.16) ^a,A^	2.38 (1.05) ^b,B^	1.78 (0.62) ^a,B^	˂0.001
35% CP	0.29 (0.19) ^a,A^	2.58 (1.24) ^b,B^	2.41 (1.15) ^a,B^	˂0.001
40% HP	0.21 (0.07) ^a,A^	3.58 (0.79) ^b,B^	1.77 (1.03) ^a,B^	˂0.001
*p*-value	0.206	˂0.001	0.050	

## Data Availability

The authors confirm that the data supporting the findings of this study are available within the article.
